# The Mediating Role of Bullying and Victimisation on the Relationship Between Problematic Internet Use and Substance Abuse Among Adolescents in the UK: The Parent–Child Relationship as a Moderator

**DOI:** 10.3389/fpsyt.2021.493385

**Published:** 2021-10-28

**Authors:** Muthanna Samara, Adeem Ahmad Massarwi, Aiman El-Asam, Sara Hammuda, Peter K. Smith, Hisham Morsi

**Affiliations:** ^1^Department of Psychology, Kingston University London, Kingston upon Thames, United Kingdom; ^2^Department of Social Work, Ben-Gurion University of the Negev, Beersheba, Israel; ^3^Department of Psychology, Goldsmiths University of London, London, United Kingdom; ^4^National Centre for Cancer Care and Research (NCCCR), Hamad Medical Corporation (HMC), Doha, Qatar

**Keywords:** parent-child relationship, parenting, victimisation, bullying, cyberbullying, addiction, problematic internet use, substance (ab)use (drugs, alcohol, smoking)

## Abstract

Over the last decade, research into the negative effects of problematic internet use has greatly increased. The current study adopted a mediation-moderation model in exploring the relationship between problematic internet use and substance abuse (drinking, drug use, and smoking tobacco cigarettes) among 1,613 adolescents (aged 10–16) in the UK. The findings of the study revealed a significant positive correlation between problematic internet use and substance abuse, which is mediated by traditional and cyber bullying and victimisation. Furthermore, the parent–child relationship was found to be a protective factor that moderated the correlation between problematic internet use and substance abuse and the correlation between problematic internet use and traditional bullying. The study emphasises the critical need to reduce problematic internet use among adolescents as a risk factor for involvement in bullying as perpetrators and victims, in addition to substance abuse. Furthermore, the findings of the study highlight the importance of a good parent–child relationship as a protective factor among adolescents. In light of the findings of the study, interventions for reducing problematic internet use taking into account bullying and the parent–child relationship are needed among adolescents.

## Introduction

The Internet has become an integral part of adolescent's lives as a communication tool for establishing relationships and participating in social groups (1). However, there are some risks and problems that adolescents may face while using the internet (2, 3). Problematic Internet Use (PIU) is described as a general behavioural addiction, which refers to cognitive preoccupation with the internet, psychological dependence on it, and an inability to control time spent on the network (4, 5). Research studies indicated that PIU is a growing problem among adolescents (6). A study of 11,956 adolescents from 11 different countries showed that the average prevalence of PIU is 4.4% (7), while it is 5.2% amongst British adolescents (aged 11–18) (8). Previous studies showed that PIU is correlated with a broad array of adverse social and psychological outcomes, such as depression, bullying, drinking and drug use (9–12).

### Problematic Internet Use (PIU) and Substance Abuse: The Mediating Role of Bullying Involvement

Problem Behaviour Theory (13, 14) suggests that problem behaviours tend to correlate and co-occur among adolescents. In other words, adolescents that are involved in one problem behaviour would be more likely to be involved in others. In line with this theory, several studies revealed a positive association between PIU and substance use among adolescents (15, 16). For example, a study conducted among 3,067 adolescents in Switzerland found that problematic internet use is an important predictor of substance use, including tobacco smoking, drinking alcohol, and consumption of drugs (17). In a similar vein, a study of 1,325 Italian adolescents (aged 11–13) found a positive correlation between problematic social networking usage and substance use (18).

While previous studies emphasised the direct association between problematic internet use and substance use, to the best of our knowledge, no research to date has explored the mediating effect of bullying and victimisation on the relationship between PIU and substance use. Bullying is defined as a specific type of aggressive behaviour that is intentional, repeated over time, and involves an imbalance of power between the bully and the victim (19). Bullying can be physical (e.g., hitting, pushing, and kicking), verbal (e.g., name calling, teasing), or relational (e.g., spread rumours, gossiping). In addition to these traditional types, bullying can also take place in electronic contexts (e.g., email, cell phones, text messages, and internet sites), which is defined as “cyber” bullying (20). Prior studies showed a significant correlation between PIU and involvement in bullying and victimisation (5, 12, 21, 22). For example, a study conducted among 6,237 Hungarian middle school adolescents revealed a significant association between PIU and involvement in traditional and cyber bullying as perpetrators and victims (23).

The few studies that examined the correlation between involvement in bullying and substance use have consistently found a link between involvement in bullying and substance use (24–27). For instance, a study conducted among middle-school adolescents in Florida found that students involved in different types of bullying as perpetrators or victims were significantly more likely to be engaged in substance use than those not involved in bullying (28).

According to Agnew's General Strain Theory (29), involvement in bullying is one type of strain that increases the likelihood of involvement in crime and anti-social behaviours (e.g., substance use), as a way to cope with the negative emotions that result from the strain.

Based on these theories (14, 29), we assume that adolescents who use the internet in problematic ways are at higher risk for involvement in bullying and/or victimisation, which may in turn lead to substance use (30, 31).

### The Moderating Role of Parent–Child Relationship

Problem Behaviour Theory (14) focused on the social perceived environment (e.g., parental factors) as a protective factor that could have a buffer effect on risky behaviours. The theory suggests that adolescents who have healthy positive relationships with their families are expected to maintain fewer problematic and risky behaviours. Previous studies showed a negative association between positive parenting and risky behaviours (e.g., problematic internet use and substance use) (32–35). For example, a study of 4,925 adolescents from France and the UK (aged 15–16) found that adolescents who were not satisfied with their relationships with their parents were more likely to be heavy substance users than others (36). Another study of 3,662 high school students in Taiwan found that a conflictual parent–child relationship was linked positively with both problematic internet use and substance use (37).

Prior studies also showed a negative association between healthy parent–child relationships and involvement in bullying as perpetrators and victims (32, 38–40). For instance, a study conducted among school students (aged 13–17) revealed that all adolescents that were victims of bullying had lower levels of social connexions with their parents (41). A meta-analysis study also found that victimisation was related to higher negative parenting (abuse and neglect, maladaptive parenting, and overprotection) and lower positive parenting (authoritative, communication, parental involvement and support, supervision, warmth, and affection) (39). In healthy and positive parent–child relationships, children tend to share their experiences of bullying with their parents and ask them for help, which protects them from further involvement in bullying (32).

Although previous studies focused on the direct effect between PIU, substance abuse, and parental factors, to the best of our knowledge, no study has explored the moderating effect of the parent–child relationship on this association, including bullying and victimisation as mediators.

### Aims of the Study

**Figure 1:**
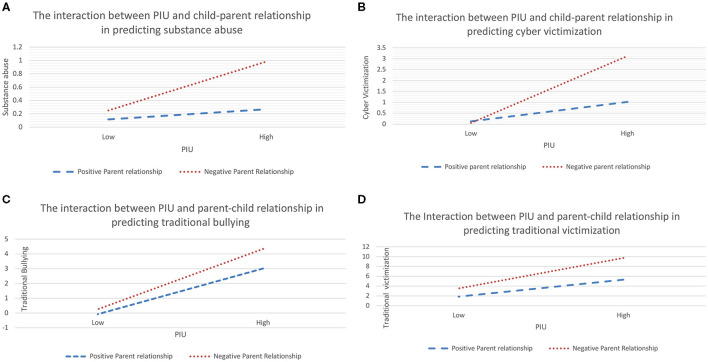
**(A)** Simple slope analysis shows that parent–child relationship moderated the relation between PIU and substance abuse. The function was graphed for two levels of independent variable and moderator: 1 SD above the mean and 1 SD below the mean. **(B)** Simple slope analysis shows that parent–child relationship moderated the relation between PIU and cybervictimisation. The function was graphed for two levels of independent variable and moderator: 1 SD above the mean and 1 SD below the mean. **(C)** Simple slope analysis shows that parent–child relationship moderated the relation between PIU and traditional bullying. The function was graphed for two levels of independent variable and moderator: 1 SD above the mean and 1 SD below the mean. **(D)** Simple slope analysis shows that parent–child relationship moderated the relation between PIU and traditional victimisation. The function was graphed for two levels of independent variable and moderator: 1 SD above the mean and 1 SD below the mean.

The current study examines the relationship between PIU and substance abuse (including drug use, smoking tobacco cigarettes, and drinking alcohol) among adolescents in the UK, and explores whether this association is mediated by involvement in traditional and cyber bullying and victimisation. Furthermore, we will examine the moderating effect of the parent–child relationship on the relationship between PIU and involvement in bullying, and the relationship between involvement in bullying and substance abuse (see Figure 1).

## Materials and Methods

### Design and Participants

A cross-sectional research design was adopted. In total, six public secondary schools across the UK agreed to take part in the study, representing northern and central UK. The original sample included 1,969 participants; however, 155 questionnaires were deemed invalid or incomplete. The final sample that included valid data for the main variables consisted of 1,613 participants who completed all related variables. Independent *t*-test analysis showed that in comparison to those who filled the full questionnaire, the participants that have not completed the question on substance abuse and thus were not included in the final analysis were more likely to be involved in cyber victimisation (Incomplete: *N*: 117; *M* = 2.74, *SD* = 8.10; complete: *M* = 0.84, *SD* = 2.80, *p* < 0.05), cyber bullying (Incomplete: *N*: 135; *M* = 2.16, *SD* = 7.98; complete: *M* = 0.31, *SD* = 2.09, *p* < 0.05), traditional victimisation (Incomplete: *N*: 135; *M* = 8.04, *SD* = 12.35; complete: *M* = 4.46, *SD* = 6.82, *p* < 0.01), and traditional bullying (Incomplete: *N*: 127; *M* = 5.39, *SD* = 11.82; complete: *M* = 1.68, *SD* = 4.08, *p* < 0.01). On the other hand, those who filled the question on substance abuse were more likely to have higher total internet use score (Complete: *M* = 8.38, *SD* = 8.62; incomplete: *N*: 20; *M* = 3.55, *SD* = 5.10, *p* < 0.001) and positive parent–child relationships (complete: *M* = 5.26, *SD* = 1.12; incomplete: *N*: 156; *M* = 4.86, *SD* = 1.41, *p* < 0.01). The participants age ranged from 10 to 16 years (*M* = 12.6, *SD* = 1.3); 53% males (*M* = 12.5, *SD* = 1.0) and 47% females (*M* = 12.7, *SD* = 1.4). All participants completed structured anonymous self-report questionnaires (hardcopy or an online version of the questionnaires). Most of the participants were white British (83.2%), 7.6% were (mostly Southern Asia), and the rest (9.2%) are non-British of different ethnic groups (e.g., Black African, White Europeans, Mixed, etc.). In addition, the participants were mostly living with both parents (75.7%), while 16.7% were living only with the mother, 1.8% only with the father and 5.8% with other people (e.g., mother and stepdad, grandparents, etc.). The mean number of siblings was 1.79 (SD: 1.37).

### Measurements

#### Parent–Child Relationship

Participants were asked to evaluate their relationship with their mothers and fathers (How would you describe your relationship with your mother; How would you describe your relationship with your father?) ranging from not good at all (0) to very good (3). The variable was constructed by adding up the two items and thus reflected the relationship with one or both parents.

#### Problematic Internet Use (PIU)

This questionnaire included 15 items (α = 0.89) adapted from Demetrovics et al. (42), and included three subscales: *Obsession* (six items, α = 0.82) (e.g., How often do you daydream about the Internet?); *Neglect* (six items, α = 0.80) (e.g., How often do you neglect school work to spend more time online?); and *Control Disorder* (three items, α = 0.67) (e.g., How often do you try to limit the amount of time spent online?). Responses ranged from never (0) to most days (4). Total Problematic Internet Use (PIU) constituted the sum of the 15 items. The total PIU and its subscales were validated using the same sample [see (9)]. In this study, the total PIU will be used.

#### Substance Use and Cigarette Smoking

This variable was assessed using a Brief Screening Test for Adolescent Substance Abuse using six Yes\No questions (α = 0.85) adapted from the CRAFT Screening Interview (43). Participants were asked to report their experiences with alcohol and drug use (1. Have you ever ridden a car driven by someone (including yourself) who was “high” or had been using alcohol or drugs?; 2. Do you ever use alcohol or drugs to relax, feel better about yourself, or fit in?; 3. Do you ever use alcohol or drugs while you are by yourself, or alone?; 4. Do you ever forget things you did while using alcohol or drugs?; 5. Do your family or friends ever tell you that you should cut down on your drinking or drug use?; 6. Have you ever gotten into trouble while you were using alcohol or drugs?). In addition, one question was regarding smoking cigarettes (tobacco). Participants were asked about the frequency of smoking cigarettes. Responses for this question ranged from never smoked (0) to more than five times a week (4). The frequency of smoking was recoded into two categories; never smoked vs. smoked. Then the seven questions were added up to form a total substance abuse variable.

#### Bullying and Victimisation

This was assessed using 16 bullying items (total traditional and cyber bullying: α = 0.93) and 16 items about victimisation (total traditional and cyber victimisation: α = 0.91).

##### Traditional Bullying and Victimisation

This was assessed using eight items for bullying and eight items for victimisation from the Olweus Bullying Questionnaire (44).

Participants were asked to indicate how many times they had bullied others in the last 6 months. Four items were related to direct bullying (e.g., hit, kicked, pushed, or threatened someone; called someone bad or nasty names) and four items were related to relational bullying (e.g., told someone I did not want to be their friend anymore; I excluded someone from groups and activities). These eight items were put together to construct a *traditional bullying variable* (α = 0.92).

Participants were also asked to indicate how many times they had experienced bullying from others as victims, in the last 6 months. Four items were related to direct victimisation (e.g., I was hit, kicked, or threatened; I was tricked in a nasty way) and four items were related to relational victimisation (e.g., other children told lies or nasty storeys about me; I was excluded from groups and activities). These eight items were put together to construct a *traditional victimisation variable* (α = 0.91).

##### Cyber Bullying and Victimisation

This was assessed using items from Smith et al. (45). Participants were asked whether they send or receive rude, offensive, cruel, or mean messages, pictures, video and comments through text messages, iMessages (e.g., WhatsApp), Emails, phone/mobile or video calls (e.g., Skype), Chat rooms (e.g., normal chat rooms, games chat rooms), Websites, Social Networks (e.g., Facebook, Twitter), or other. This could take the form of bullying others (being a bully) (α = 0.96) or being bullied by others (α = 0.92).

Responses for both traditional and cyber bullying items ranged from 0 (never) to 4 (several times a week).

### Ethical Consideration

The study was approved by the Ethical Committee of Kingston University London, U.K., according to the British Psychological Society's ethical standards and regulations. All parents gave written informed consent for their children and adolescents to participate in the study.

### Procedure

Following ethical approval from Kingston University London, schools were sent parental consent forms and obtained an agreement from the entire sample. The questionnaires were available either online (in a designated school IT room) or as a hardcopy in the classroom; 70% of the children completed the online questionnaire (*via* Qualtrics.com) and 30% completed the hardcopy. In both cases, the researcher gave instructions and help on how to fill in the questionnaires. Children were told that participation was voluntary and that they could withdraw at any time without explanation. Children were encouraged to provide as accurate information as possible and to talk to the school counselling team if they felt uncomfortable because of their participation.

### Data Analysis

First, descriptive statistics were examined regarding the variables of the study. Second, bivariate analyses were conducted to test the relationships between the research variables using Pearson's correlations. Third, we performed a PROCESS mediation-moderation analysis using SPSS 26 [PROCESS-Model #59 developed by Preacher and Hayes (46)], which simultaneously explores mediation and moderation, to test the mediating role of bullying and victimisation (traditional and cyber) on the relationship between total PIU and substance abuse (model 4). In addition, we explored the moderating effect of the child–parent relationship on three paths: The direct relationship between PIU and substance abuse; the relationship between PIU with the mediators (traditional and cyber bullying and victimisation), and the relationship between the mediators (bullying and victimisation) and substance abuse (model 59).

The mediation path and the direct effects are assumed to be moderated by the child–parent relationship. The 95% confidence interval obtained with 1,000 bootstrap resamples was used (46). Once a bootstrap sample of the original data is generated, the regression coefficients for the statistical model are estimated. This procedure yields an upper and lower bound of the confidence interval on the likely value of the indirect effect for the three cut points of the moderating factor (mean, +-SD). If the confidence interval does not straddle zero, this leads to the inference that the indirect effect is not zero and that there is a significant mediation. The means of all the variables were centred.

## Results

### Descriptive Statistics

The descriptive statistics for each variable (mean, s.d.) are given in Table 1. Regarding the items of substance abuse, 8% of the adolescents reported that they rode in a car driven by someone who had been using alcohol or drugs, 4.9% reported that they used drugs or alcohol to relax or feel better about themselves, 3.6% reported that they used drugs or alcohol while they are alone, 3.1% reported that their families or friends tell them that they should cut down on their drinking or drug use, and 3.8% reported they gotten in trouble while they are using alcohol or drugs. In addition, 2.7% of adolescents reported that they smoke cigarettes 1–5 times in the last 6 months, 0.6% 1–5 times a month, 0.6% 1–5 times a week, and 1.4% more than 5 times a week. This was categorised into smoked a cigarette at least once in the last 6 months (5.2%) vs. none. Substance abuse was then constructed of the above seven items (six on drugs and alcohol and one on smoking) ranging from 0 to 7; the higher the number the more frequent the substance abuse behaviour.

**Table 1:** Descriptive statistics and correlations among the main variables (*N* = 1,613).

**Variable**	**Mean**	**SD**	**1**	**2**	**3**	**4**	**5**	**6**	**7**
1. Substance abuse	0.331	1.07	1						
2. Total PIU	8.32	8.60	0.199*	1					
3. Traditional bullying	1.94	5.14	0.355**	0.344**	1				
4. Cyber bullying	0.42	2.86	0.364**	0.238**	0.644**	1			
5. Traditional victimisation	4.73	7.44	0.261**	0.280**	0.617**	0.533**	1		
6. Cyber victimisation	0.97	3.44	0.310**	0.302**	0.577**	0.806**	0.653**	1	
7. Parent–child relationship	5.23	1.15	−0.152**	−0.117**	−0.217**	−0.149**	−0.265**	−0.216	1

*P < 0.05; **P < 0.01.

The independent samples *t*-test showed that those who do not live with both parents (mother only, father only or other) were more likely to be involved in substance abuse behaviour [*t*_(1, 622)_ = −2.78, *p* < 0.01], to be traditional victims [*t*_(1, 748)_ = −2.67, *p* < 0.01] and cyber victims [*t*_(1, 726)_ = −3.65, *p* < 0.001], and to have negative parent–child relationship [*t*_(1, 761)_ = −12.09, *p* < 0.001].

### Bivariate Analysis: Relationship Between PIU, Substance Abuse, and Bullying/Victimisation

The correlational findings in Table 1 show that total PIU was significantly correlated positively with substance abuse, bullying, and victimisation (traditional and cyber). In addition, bullying and victimisation (traditional and cyber) were correlated positively with substance abuse. On the other hand, parent–child relationship was significantly correlated negatively with substance abuse, PIU total, bullying, and victimisation (except for cybervictimisation) (see Table 1).

### Mediation Effect Analysis

Model 4 is a simple mediating model in the SPSS macro PROCESS compiled by Hayes (47). This was adopted to test the mediating effect of bullying/victimisation on the relationship between PIU and substance abuse. The results are shown in Table 2. Model 1 of Table 2 shows that the positive predictive effect of PIU on substance abuse was significant (B = 0.02, *t* = 7.49, *p* < 0.001). Model 2 of Table 2 shows that PIU had a significant positive predictive effect on both forms of bullying and victimisation (traditional and cyber). In turn, bullying and victimisation (both forms) had a significant positive predictive effect on substance abuse. Moreover, when mediating variables were added, the direct predictive effect of PIU on substance abuse was still significant, as shown in Model 3 of Table 2 (see Table 2).

**Table 2:** Regression model summary of the mediating effect of bullying/victimisation (traditional and cyber) on the relationship between PIU and substance abuse (Model 4) (*N* = 1,613).

**Predictors (IV)**	**Model 1 (DV: substance use)**			**Model 2 (DV: traditional bullies)**			**Model 3 (DV: substance use)**		
	**B**	**SE**	***t***	**B**	**SE**	***t***	**B**	**SE**	***t***
PIU	0.02***	0.003	7.49	0.16*	0.01	14.78	0.011***	0.003	3.51
Traditional bullies							0.089***	0.007	13.06
*R* ^2^	0.034			0.12			0.131		
*F*	56.18***			218.52***			121.96***		
				**Model 2 (DV: cyberbullies)**			**Model 3 (DV: substance use)**		
PIU				0.06***	0.006	9.78	0.013***	0.003	4.33
Cyberbullies							0.17***	0.012	14.24
*R* ^2^				0.057			0.144		
*F*				95.73***			133.11***		
				**Model 2 (DV: traditional victimisation)**			**Model 3 (DV: substance use)**		
PIU				0.21***	0.018	11.87	0.017***	0.003	5.48
Traditional victimisation							0.036***	0.004	8.98
*R* ^2^				0.079			0.085		
*F*				141.05***			75.33***		
				**Model 2 (DV: cybervictimisation)**			**Model 3 (DV: substance use)**		
PIU				0.099***	0.008	12.91	0.014***	0.003	4.64
Cybervictimisation							0.107***	0.009	11.28
*R* ^2^				0.093			0.109		
*F*				166.66***			99.63***		

*P < 0.05; ***P < 0.001.

In addition, the upper and lower bounds of the bootstrapped 95% CI for the direct effect of PIU on substance abuse and the mediating effect of bullying and victimisation (both forms) did not include 0, indicating that the mediating effect was significant [Traditional bullying: indirect effect = 0.014, SE = 0.004, 95% CI = (0.005, 0.023); Cyberbullying: indirect effect = 0.010, SE = 0.005, 95% CI = (0.002, 0.019); Traditional victimisation: indirect effect = 0.008, SE = 0.003, 95% CI = (0.003, 0.014); Cybervictimisation: indirect effect = 0.011, SE = 0.004, 95% CI = (0.004, 0.019)]. Of the total effect, the mediation effect accounted for 13.2% for traditional bullies, 14.4% for cyberbullies, 8.5% for traditional victims, and 10.9% for cybervictimisation, which suggests that bullying and victimisation played a partial mediating role in the relationship between PIU and substance abuse.

### Moderated Mediation Effect Analysis

To test the moderated mediation model, we used Model 59 of the SPSS macro PROCESS compiled by Hayes (47). The results of the child–parent relationships moderation test are shown in Table 3. After putting child–parent relationships into the model, the product (interaction term) of PIU and child–parent relationships had a significant negative predictive effect on substance abuse, as shown in Model 1 of Table 3 (B = −0.011, *t* = −5.28, *p* < 0.001) (see Table 3 and Figure 1A). In addition, the interaction term of PIU and child–parent relationships had a significant negative predictive effect on traditional bullying and victimisation and cybervictimisation (but not on cyberbullies), as shown in Model 2 of Table 3 (see Table 3 and Figures 1B–D).

**Table 3:** Regression model summary of the moderation-mediation model predicting substance abuse.

**Predictors (IV)**	**Model 1 (DV: substance use)**			**Model 2 (DV: traditional bullies)**			**Model 3 (DV: substance use)**		
	**B**	**SE**	***t***	**B**	**SE**	***t***	**B**	**SE**	***t***
PIU	0.02***	0.003	7.15	0.15***	0.01	13.99	0.009**	0.003	2.99
Parent–child relationships	−0.10***	0.023	−4.57	−0.34***	0.08	−4.02	−0.083***	0.022	−3.72
PIU × Parent–child relationships	−0.011***	0.002	−5.28	−0.033***	0.007	−4.34	−0.012***	0.003	−4.95
Traditional bullies							0.081***	0.007	11.70
Traditional bullies × parent–child relationships							0.010**	0.004	2.66
*R* ^2^	0.075			0.148			0.149		
*F*	43.84***			92.483***			55.74***		
				**Model 2 (DV: cyberbullies)**			**Model 3 (DV: substance use)**		
PIU				0.06***	0.005	10.18	0.012***	0.003	3.98
Parent–child relationship				−0.022	0.046	−0.47	−0.099***	0.022	−4.49
PIU × Parent–child relationships				0.002	0.005	0.546	−0.010***	0.002	−4.24
Cyberbullies							0.160***	0.012	13.25
Cyberbullies × Parent–child relationship							0.004	0.01	0.32
*R* ^2^				0.063			0.155		
*F*				35.16***			57.82***		
				**Model 2 (DV: traditional victimisation)**			**Model 3 (DV: substance use)**		
PIU				0.19***	0.018	10.67	0.015***	0.003	5.04
Parent–child relationships				−1.15***	0.14	−8.27	−0.076**	0.023	−3.24
PIU × Parent–child relationships				−0.038**	0.002	−4.88	−0.011***	0.002	−4.88
Traditional victimisation							0.031***	0.004	7.48
Traditional victimisation × Parent–child relationships							0.003	0.002	1.34
*R* ^2^				0.128			0.107		
*F*				79.04***			38.45***		
				**Model 2 (DV: cybervictimisation)**			**Model 3 (DV: substance use)**		
PIU				0.090***	0.008	11.85	0.013***	0.003	4.28
Parent–child relationships				−0.190**	0.060	−3.18	−0.091***	0.023	−4.04
PIU × Parent–child relationships				−0.034***	0.005	−6.30	−0.010***	0.002	−4.26
Cybervictimisation							0.096***	0.010	9.68
Cybervictimisation × Parent–child relationships							0.010	0.005	1.88
*R* ^2^				0.128			0.127		
*F*				78.61***			46.69***		

The mediation effect of bullying and victimisation (traditional and cyber) on the relationship between PIU and substance abuse and the effect of parent-child relationship as a moderator (Model 59) (N = 1,613). **P < 0.01; ***P < 0.001.

Model 3 of Table 3 shows that only traditional bullying and parent–child relationships interaction had a significant predictive effect on substance abuse (B = 0.01, *t* = 2.66, *p* < 0.01).

**Figure 2:**
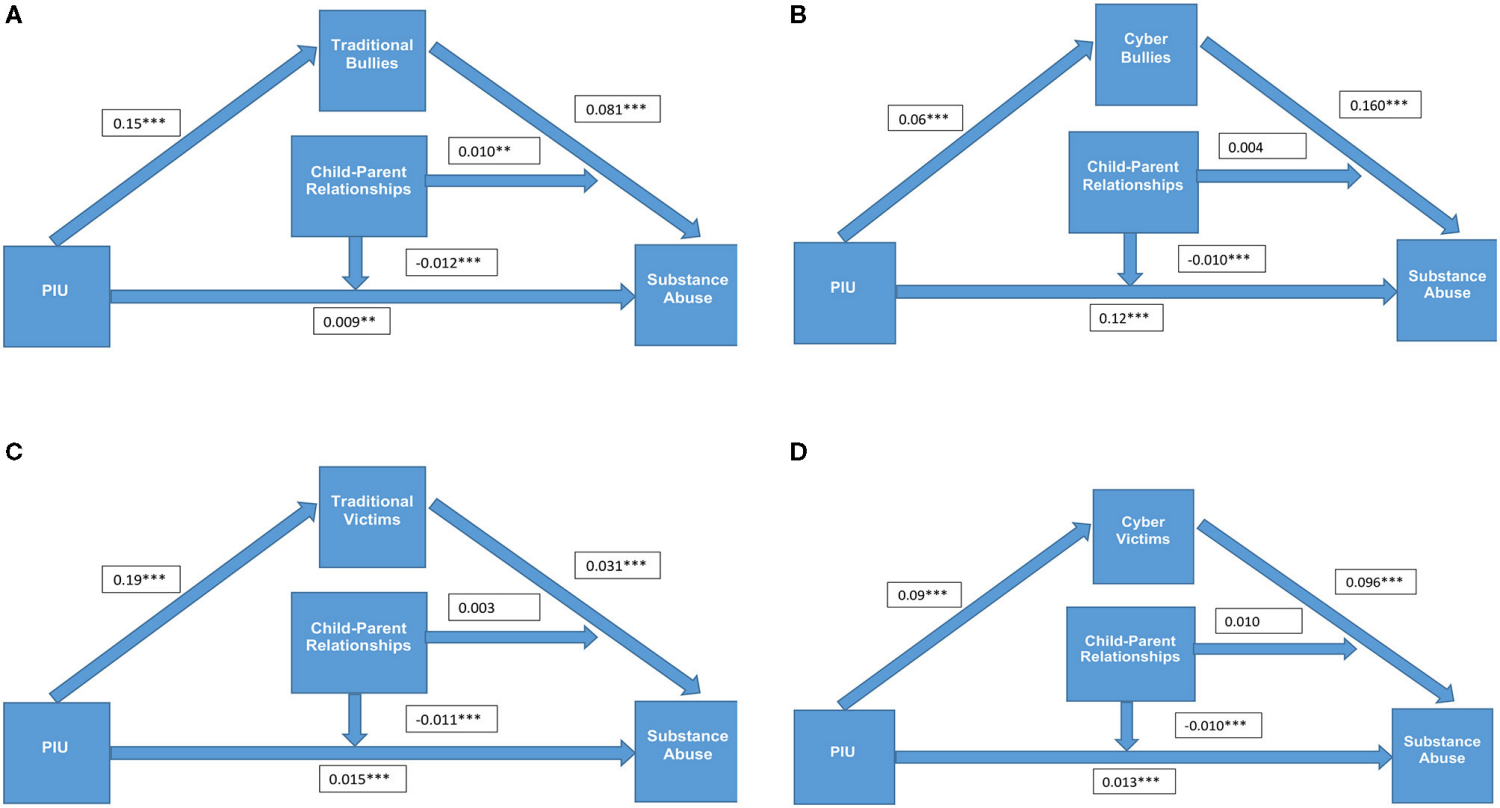
**(A)** The moderated mediation model when traditional bullies is the mediator. **(B)** The moderated mediation model when cyber bullies is the mediator. **(C)** The moderated mediation model when traditional victims is the mediator. **(D)** The moderated mediation model when cyber victims is the mediator. **P < 0.01; ***P < 0.001.

These results suggest that parent–child relationships played a moderating role in the relationships between PIU and substance abuse (model 1), between PIU and bullying and victimisation (except for cyberbullies) (model 2), and between traditional bullying and substance abuse (model 3) (see Figures 2A–D).

This indicates that for individuals with low levels of parent–child relationships, higher levels of PIU were associated with higher levels of substance abuse. In addition, bullying significantly predicted substance abuse in low-level child–parent relationships.

Of the total effect, the mediation moderation effect accounted for 14.9% for traditional bullies, 15.5% for cyberbullies, 10.7% for traditional victims, and 12.7% for cybervictimisation, which suggests that bullying and victimisation and the interaction with child–parent relationships played a partial mediating role in the relationship between PIU and substance abuse.

## Discussion

The current study explored the mediation effect of involvement in traditional and cyber bullying and victimisation on the relationship between PIU and substance abuse amongst adolescents in the UK. The findings of the study revealed that the correlation between PIU and substance abuse among adolescents is partially mediated by involvement in bullying as perpetrators and victims. In addition, the parent–child relationship was specifically found as a significant moderator on the relationship between PIU and traditional bullying and victimisation and cybervictimisation. It has also moderated the relationship between traditional bullying and substance abuse.

### Involvement in Bullying and Victimisation as Mediators Between PIU and Substance Abuse

In accordance with previous studies (15, 16, 48), the findings of the current study indicated that adolescents who reported higher levels of PIU were more likely to be engaged in substance abuse. This finding is in line with the Problem Behaviour Theory (14) that suggests that risky behaviours can co-occur and that adolescents who are engaged in one risky behaviour are more likely to be involved in other risky behaviours. This may also suggest that the two behaviours are similar because they both may lead to addiction. It is of interest that addictive behaviours concerning both drug usage and non-drug usage are related to neuro-bio-cognitive disruption of one or a combination of three key neural systems that are responsible for willpower. The proposed model suggests a disruption of the impulsive amygdala regulated system, the reflective prefrontal regulated system and/or the feeling insula regulated system (49). It is possible that the lack of control of risky PIU, substance abuse, and bullying behaviours are also related to this neuro-bio-cognitive impairment and future research ought to investigate this.

In addition to this direct correlation, the findings of the current study showed that the correlation between PIU and substance abuse is partially mediated by involvement in traditional and cyber bullying. Adolescents who spend more time on the internet were at greater risk for involvement in bullying as perpetrators and victims, which in turn increases their likelihood to be involved in substance abuse. Those who are unable to control their internet use could have difficulty in controlling their behaviours as well, which as a result may increase the risk for acting aggressively (24, 50).

We can understand this mediation process in light of the General Strain Theory of Agnew (29). According to this theory, substance abuse among adolescents is a coping mechanism for relieving negative feelings, such as stress, frustration, and depression, caused by the strain of being involved in bullying. With limited support and skills, adolescents who are involved in bullying may resort to substance abuse and self-injury behaviours to escape and cope (51).

An additional explanation could be related to the adolescent's desire to gain social status and to be perceived as cool; and attractive. For adolescents, smoking and drinking is a behaviour that contributes to the social image of the individual and can be well-used for this purpose (52).

Consistent with previous studies, bullies may be more vulnerable to substance abuse than others (28). The findings of the study showed a positive association between involvement in bullying and substance abuse, but this correlation was stronger among bullies than victims, particularly in traditional bullying. This indicates that bullies are more susceptible to risky behaviours than victims.

Additionally, PIU includes neglect of daily activities and social interactions. This may lead to a lack of skills relating to social peer relationships and thus increase the risk of getting involved in bullying in one way or another.

### Parent–Child Relationship: Direct and Interactive Effects

In line with previous studies that emphasised the parent–child relationship as a protective factor among adolescents (36, 37, 53), the findings of the current study revealed a negative correlation between parent–child relationship in one hand and PIU and substance abuse on the other hand. Adolescents who described their relationships with their parents as positive, reported lower levels of PIU and substance abuse. We can understand the protective effect of the parent–child relationship in light of the Social Bond Theory of Hirschi (54). This theory suggests that adolescents, who are close to their parents, feel obligated to act in non-deviant ways to please their parents, so they are less likely to be involved in risky behaviours, such as substance abuse.

Our findings also showed that a good parent–child relationship serves as a moderator of the relationship between PIU with traditional bullying and victimisation and cybervictimisation (but not of cyberbullies). In addition, the parent–child relationship moderates the relationship between traditional bullying and substance abuse but not cyberbullying. These findings can be explained by the significant relationship that was found between not living with both parents on one hand and victimisation, substance abuse, and a negative parent–child relationship on the other. This indicates that those who do not live with both parents are more at risk of developing behavioural problems (55), including internalising and externalising behaviours (e.g., substance abuse) (56, 57).

To the best of our knowledge, no research to date has explored the moderating effect of the parent–child relationship on these correlations. These findings emphasise that a good relationship between adolescents and their parents protects them from involvement in risky behaviours (bullying and substance abuse), despite their involvement in other risky behaviour (such as PIU). Positive and supportive communication helps children acquire adapting coping strategies, which reduce their engagement in risky behaviours (58).

These findings are also consistent with prior research suggesting that high support and warm relationships between parents and children are most likely to protect adolescents against involvement in bullying and victimisation (39). This moderation can be explained by Regulation Theory (59), which argues that sensitive parents could help their children regulate their emotions and behaviours. When parents fail to provide enough guidance and support through communication with their children, the children will have difficulty regulating their emotions effectively, which may increase their vulnerability to being involved in bullying as means of dealing with distress (60). Through conversation and open communication, parents can help their children develop behavioural schemes based on their experiences and perceptions. This in turn could help them to cope and avoid victimisation and bullying by internalising positive conflict-solving skills (32, 40, 61). However, the findings of the study showed that only the correlation between traditional bullying and substance abuse was moderated by the parent–child relationship, but not cyber bullying and victimisation and traditional victimisation. This could be because traditional bullying is a “visible” behaviour compared to cyber bullying and victimisation, which gives parents the ability to identify problems in their children's lives and deal with them. Also, this may indicate that victims usually suffer in silence (62, 63) and thus are in need of more support. Parents need to look at possible risks and engage more with their children to be able to recognise these behaviours.

### Conclusions and Implication for Practise

The findings of the current study indicated that PIU is a major risk factor for substance abuse amongst adolescents. This is mediated by involvement in traditional and cyber bullying and victimisation. Furthermore, a good parent–child relationship was found to be an important protective factor that buffers the risk for involvement in substance abuse. This emphasises the importance of examining adolescents' behaviours in the context of their relationships and examining both risk and protective factors. To strengthen the reliability of the findings and to examine this problem from several points of view, future research needs to include additional informants such as parents and teachers. In addition, future studies should use longitudinal designs to determine cause-and-effect relationships between the independent variables and the outcome variable. In this study, not all children completed the substance abuse questionnaire and thus were excluded from the analysis. Children and adolescents who were involved in cyber and traditional bullying and victimisation were more likely to be excluded from the study. However, the participants who were included were more likely to have higher total internet use scores and positive parent–child relationships. Nevertheless, empirical evidence and simulations indicate that regression models validity is only marginally affected even after selective dropout. That is, the relation between predictors and outcome is unlikely to be substantially altered by selective dropout (64).

The substance abuse variable constitutes seven questions, which may not reflect the quantity of alcohol and drugs as the question on smoking. However, the questions reflect lifestyle rather than only the current situation and gives a good indication of being involved in risky behaviours in general. In addition, the described behaviour in question number one (Have you ever ridden a car driven by someone (including yourself) who was “high” or had been using alcohol or drugs?) may be related directly to other people's behaviour (e.g., parents, siblings, and friends) rather than directly to the respondent. Nevertheless, the question may indicate that the surrounding proximal environment of the targeted adolescent is a predisposition of toxic stress that may form a risk factor for their behaviour and physical and mental health (65). In that sense, children do not develop in isolation but develop in an environment of relationships (66, 67), and while a negative stressful environment may lead to behaviour and mental problems, supportive nurturing and safe relationships and environments, on the other hand, can buffer the response to toxic stress and thus lead to improved outcomes of physical and mental health (68, 69).

Therefore, we recommend that future research explores additional aspects of parenting, such as parenting styles and parental involvement to understand the exact parental behaviour that can affect these relationships. In addition, it is necessary to explore risk factors for problematic internet use among adolescents, at the level of the individual, the family, and the social context.

The findings of the study have several implications for practise. In light of the findings, it is important that mental health and psychology professionals develop programs for preventing problematic internet use among adolescents in addition to behavioural interventions with adolescents who use the internet in problematic ways. Professionals need to take into account bullying and victimisation as possible mediators for the relationships between problematic internet use and substance abuse (70, 71). In addition, practitioners who work with adolescents should include parents in their intervention programs with the aim of improving parent–adolescent relationships. Interventions may also include improving peer and sibling relationships (72, 73) and face-to-face or online therapies (74).

## Data Availability Statement

The dataset for this manuscript are not publicly available because they are used in other ongoing studies for publication. Requests to access the datasets should be directed to the corresponding author of the manuscript (Muthanna Samara: m.samara@kingston.ac.uk).

## Ethics Statement

The studies involving human participants were reviewed and approved by the Ethical Committee, Kingston University London, UK. Written informed consent to participate in this study was provided by the participants' legal guardian/next of kin.

## Author Contributions

MS, PS, and HM contributed to the conception and design of the study. MS, AE-A, AM, and SH organised the database, performed the statistical analysis, contributed to the acquisition, and interpretation of data for the work. All authors drafted the work and revised it critically for important intellectual content and approved the submitted version.

## Funding

This study was supported by Qatar National Research Fund (QNRF) a member of Qatar Foundation Doha, Qatar, National Priority Research Programs (NPRP) under Grant (NPRP 5 - 1134 - 3 - 240).

## Conflict of Interest

The authors declare that the research was conducted in the absence of any commercial or financial relationships that could be construed as a potential conflict of interest.

## Publisher's Note

All claims expressed in this article are solely those of the authors and do not necessarily represent those of their affiliated organizations, or those of the publisher, the editors and the reviewers. Any product that may be evaluated in this article, or claim that may be made by its manufacturer, is not guaranteed or endorsed by the publisher.
